# Preconception Dietary Patterns and Associations With IVF Outcomes: An Ongoing Prospective Cohort Study

**DOI:** 10.3389/fnut.2022.808355

**Published:** 2022-02-16

**Authors:** Shanshan Wu, Xudong Zhang, Xinyang Zhao, Xinyao Hao, Siwen Zhang, Pingping Li, Jichun Tan

**Affiliations:** ^1^Department of Obstetrics and Gynecology, Center of Reproductive Medicine, Shengjing Hospital of China Medical University, Shenyang, China; ^2^Key Laboratory of Reproductive Dysfunction Disease and Fertility Remodeling of Liaoning Province, Shenyang, China

**Keywords:** preconception, dietary patterns, principal components analysis, cluster analysis, IVF

## Abstract

There is a lack of research on preconception diet and reproductive outcomes conducted in the Chinese population using individual assessment. Between April 2017 and April 2020, 2,796 couples undergoing *in vitro* fertilization treatment were recruited in this ongoing prospective cohort, and 1,500 eligible couples were included in the final analysis. A validated semi-quantitative food frequency questionnaire was used to evaluate the maternal preconception diet. Other lifestyle factors, including smoking status, psycho-mental status, sleep quality, and physical activity, were also assessed. Five dietary patterns were identified using principal component analysis, namely “Fruits-Vegetables-Dairy-Eggs,” “Fish/Seafood-Animal blood,” “Tubers-Beans-Cereals,” “Puffed food-Candy-Bakery,” and “Dried Fruits-Organs-Rice.” After adjusting for multiple confounders, we detected that the women who are more inclined to the “Fruits-Vegetables-Dairy-Eggs” pattern and less adherent to the “Tubers-Beans-Cereals” were more likely to achieve normally fertilized eggs and transferable embryos. Regarding pregnancy outcomes, we observed that a lower “Puffed food-Candy-Bakery” score and a higher “Dried fruits-Organs-Rice” score were related to a higher likelihood to achieve biochemical pregnancy. In terms of pregnancy complications, an inverse association between “Fish/Seafood-Animal blood” and hypertensive disorders was observed. We further clustered the dietary patterns based on the proportion of food groups consumed and found that dairy intake was beneficial to embryo quality, while frequent rice consumption was associated with a higher risk of macrosomia. Notably, in the stratified analysis, we observed that the positive relationship between the “Fruit-Dairy-Vegetables-Eggs” score and normal fertilization and the inverse association of the “Fish/Seafood-Animal blood” score with hypertensive disorders during pregnancy were exhibited only among women with body mass index ≥25 kg/m^2^. In conclusion, pre-treatment diets might be an important target for intervention to achieve a better reproductive outcome.

## Introduction

Infertility is the inability to conceive within 12 months of regular, unprotected sexual intercourse (1), which has been recognized as a public health issue worldwide by the World Health Organization (WHO). The global disease burden of infertility has been increasing since 1999, estimated to affect about 10–15% of women of reproductive age (2–4). Although *in vitro* fertilization (IVF) treatment is a common alternative, the financial burden of treatment has deterred many couples (5). As a result, emerging scientific studies have been devoted to identifying modifiable factors which may affect human fertility, including diet, physical activity, stress, and lifestyle (6–9).

Studies on the relationship between micronutrients and fertility suggested that nutrition affects reproductive health (10–14), among which, folic acid and vitamin D are widely concerned. Compared with individual nutrients, the dietary pattern can represent the comprehensive effect of all foods consumed in one's diet. Therefore, research on dietary patterns provides a broader view of food and nutrient intake and overcomes the methodological limitations of studying a single nutrient in food (15, 16). In addition, previous studies have suggested that dietary patterns are relatively stable from preconception to pregnancy (17). Several studies have reported that maternal dietary patterns may be associated with reproductive outcomes, such as ongoing pregnancy, pregnancy complications, preterm delivery, and neonatal defect (18–26). Thus, establishing a healthy habitual diet before pregnancy may be an important intervention target to improve reproductive health.

There are limited pieces of evidence on the association between dietary patterns and IVF outcomes (27–35). Among these studies, the “Mediterranean diet (MedDiet)” is a hot topic (29, 31, 33–35), while few studies focus on the analysis of diversified dietary patterns (27, 28, 30, 32). A study conducted by Vujkovic et al. (28) recruited 161 couples undergoing IVF treatment and identified two dietary patterns by principal component analysis (PCA), namely “health conscious–low processed” and “Mediterranean” diet (28). It was reported that high adherence by the couple to the “Mediterranean” diet increased the probability of pregnancy (OR: 1.4, 95% CI:1.0–1.9). Another study conducted on the Rotterdam Periconceptional Cohort of 228 women with a singleton ongoing pregnancy revealed that periconceptional maternal adherence to a high fish and olive oil, low meat dietary pattern was positively associated with embryonic growth (32). Notably, the sample sizes of the abovementioned two studies were relatively limited, and other periconceptional lifestyle factors, such as psycho-mental status, sleep quality, and physical activity, have not been adjusted. Furthermore, most of the available studies were conducted in western countries, nevertheless, dietary habits are population-specific, and there are distinctive differences between Chinese and Western diets (36). Therefore, large-scale studies to explore the association between preconception diet and reproductive outcomes with adjustment of confounding bias introduced by varied lifestyle factors are warranted.

The major objective of this prospective cohort study is to assess the associations between preconception dietary patterns and reproductive outcomes following IVF treatment, ranging from embryonic development to pregnancy complications, in a large Chinese population. In this way, we may go a step further in giving proper advice on the dietary pattern for couples who prepare to have a child.

## Materials and Methods

### Study Population

Data were obtained from couples recruited in an ongoing prospective cohort study, which was conducted at the reproductive center of a university in Shenyang, China. 2,796 couples were enrolled between April 2017 and April 2020. The inclusion criteria were as follows: (1) Chinese couples settled in the northeast of China; (2) willing to cooperate to complete the questionnaires across the gestational period; (3) intended to stay in the northeast of China for at least 3 years after delivery. All participants gave their informed consent for inclusion before they participated in the study. The study was conducted in accordance with the Declaration of Helsinki, and the protocol was approved by the Ethics Committee of the Shengjing Hospital of China Medical University (2017PS269K).

**Figure 1:**
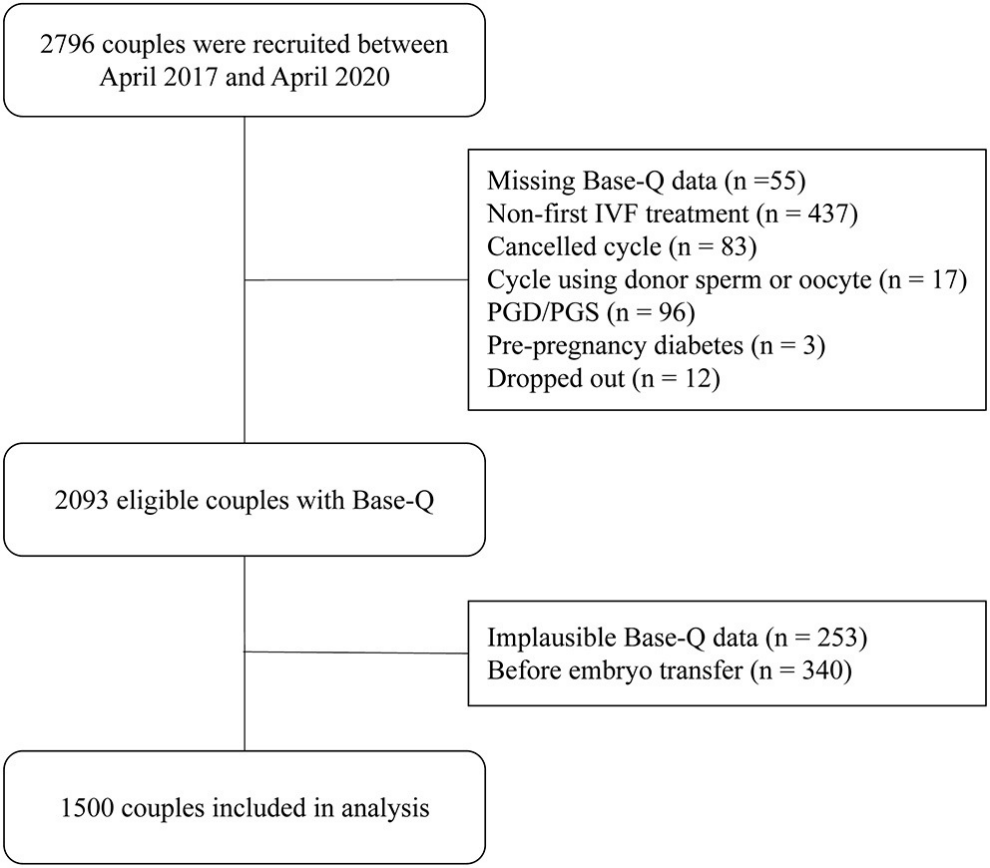
The flowchart of the selection process of this study population.

To investigate the effects of preconception dietary pattern on IVF outcomes, we only included the couples undergoing their first IVF treatment and excluded the couples: (1) using donor sperm or oocyte; (2) performing preimplantation genetic diagnosis (PGD) or preimplantation genetic screen (PGS); (3) women with pre-pregnancy diabetes. The flowchart of the selection process is presented in Figure 1.

### IVF Procedures and Outcomes Assessment

The timeline of IVF treatment was previously described in detail (37–39). Briefly, the IVF process includes the following four steps: (1) controlled ovarian hyperstimulation (COH), (2) oocyte retrieval, (3) embryo transfer, and (4) pregnancy tests. Ovulation was triggered using human chorionic gonadotropin (hCG) when there were three or more dominant follicles (diameter ≥18 mm). After 34–36 h of hCG injection, oocyte retrieval was performed under the guidance of ultrasound.

The oocytes underwent IVF or intracytoplasmic insemination (ICSI) upon clinical indication. The number of oocytes with two pronuclei and good-quality embryos formed were evaluated by embryologists. The Peter system was used to evaluate the embryo quality on day 3 (40). Embryos with more than three cells and anucleate fragmentation <50% were classified as having transferable quality. Embryos with 6–10 cells and anucleate fragmentation <20% were identified as good-quality embryos. The possibility of normal fertilization, transferable, and good-quality embryos on day 3 were considered as embryonic developmental parameters.

In general, one or two embryos were transferred on the third or fifth day after fertilization. A serum β-hCG level >30 mIU/mL on day 14 after embryo transfer was considered to indicate biochemical pregnancy. Intrauterine gestational sac confirmed by ultrasound 28 days after embryo transfer was considered clinical pregnancy. The pregnancy loss before the 12 gestational weeks (GWs) was defined as early abortion, while the miscarriage occurring after 12 GWs but before 18 GWs was defined as late abortion. Live birth was defined as the delivery of a live newborn, and preterm delivery was defined as delivery at 36^+6^ GWs or below. Neonatal birth weight <2,500 g was considered as low birth weight, while >4,000 g was considered as macrosomia. Gestational diabetes mellitus (GDM) was diagnosed during 24–28 GWs if the participant met at least one of the following criteria: (1) fasting plasma glucose ≥5.1 mmol/L, (2) 1-h plasma glucose ≥10.0 mmol/L, and/or (3) 2-h blood glucose >8.5 mmol/L. Hypertensive disorders during pregnancy include gestational hypertension, pre-eclampsia, eclampsia, chronic hypertension, and chronic hypertension with superimposed pre-eclampsia. Gestational hypertension was defined as the onset of high blood pressure (at least 140 mmHg systolic or 90 mmHg diastolic) without proteinuria on two occasions at least 4 h apart in an ordinarily normotensive pregnant woman after 20 GWs. Pre-eclampsia or eclampsia was defined as gestational hypertension with concurrent proteinuria.

### Questionnaire Collection

In this ongoing prospective cohort, both male and female partners are required to complete a questionnaire (base–Q) with 284 items on dietary intake and lifestyle before commencing ovulation induction. Furthermore, the female partners with positive β-hCG test would complete (1) the first questionnaire (T1–Q) with 219 items on medication and lifestyle, complete at the day of the B-ultrasonic pregnancy test; (2) the second questionnaire (T2–Q) with 169 items on clinical and lifestyle information during 22–26 GWs; (3) the third questionnaire (T3–Q) with 185 items on pregnancy complications and lifestyle at 30–34 GWs. The timing of the collection of questionnaires is shown in Supplementary Figure 1. Questions regarding the clinical information and IVF outcomes were obtained from electronic medical systems and completed by medical staff, while the information associating lifestyle and the dietary pattern was filled in by patients under the guidance of trained clinical staff. Internal quality control was carried out according to the audio recordings automatically obtained by the system to ensure credibility.

To explore the association between pre-treatment diet and IVF outcomes, the information on maternal lifestyle during the preconception period was extracted from base-Q. In detail, the base-Q is composed of four parts: dietary intake, micronutrient supplement, psycho-mental status assessment, and other lifestyle factors. At the end of the whole questionnaire, we designed three questions, which were repetitive with the body of the questionnaire, to preliminarily assess the retest reliability. Taking the answers in the main body of the questionnaire as the standard, if the number of correctly answered questions among these three questions is three, two, one, and zero, the reliability is assigned as 100, 67, 33%, and 0, respectively. The questionnaires with reliability below 67% were considered implausible and excluded, as shown in Figure 1.

Preconception dietary intake assessment was based on a self-administered, semi-quantitative food frequency questionnaire (FFQ). Participants were required to recall their frequency of intake and portion size consumed during the past 12 months. The FFQ was derived and modified from the validated Chinese dietary FFQ from the Chinese Center for Disease Control and Prevention (41, 42), which was widely used in various studies (43–48). Previous studies suggested that the reproducibility and validity of the Chinese dietary FFQ were satisfactory and it can be used to classify the subjects according to their food consumption in 1 year (41, 42). The FFQ adopted in this study contains 48 questions covering 24 non-overlapping food groups, which are based on nutrients, common characteristics, and culinary use, and 18 non-quantitative questions regarding beverages and spicy food intake (Supplementary Table 1). Frequency options include: (1) every day; (2) 4–6 times a week; (3) 1–3 times a week; (4) 1–3 times a month; (5) almost never/never. Amount per day or per times options include: (1) <25 g; (2) 25–50 g; (3) 50–100 g; (4) 100–150 g; (5) 150–200 g; (6) 200–300 g; (7) >300 g. Data were converted into weekly consumption by multiplying the frequency and serving per intake. The percentages of weekly intake of the food groups were calculated as consumption of each food divided by total consumption of all foods. Total energy intake for each participant was calculated based on China Food Composition Tables (49).

The consumption of micronutrient supplementation (folic acid, multivitamins, vitamin A~E, calcium, and iron) was assessed by base-Q as well. The frequency options were set as described above. We listed the common brands of supplements on the market and the corresponding content of each tablet so that the subjects can recall their daily intake as much accurately as possible. Afterward, standardized daily intake of each supplementation weighted by frequency was figured out and treated as a continuous variable in further analysis.

The stress, anxiety, and depression status of participants were assessed by the perceived stress scale, self-rating anxiety scale, and self-rating depression scale, respectively. These scales have previously been applied and validated in a Chinese population in previous studies (50–53).

Other lifestyle factors include smoking, passive smoking, sleep quality, physical activity, and work intensity. Sleep quality was evaluated by the Pittsburgh sleep quality index, which has been used in the Chinese population (54, 55). Physical activity was evaluated by the Chinese version of the International Physical Activity Questionnaire, and weekly metabolic equivalents (METs) were figured out according to the method described in previous studies (56, 57). The levels of work intensity were categorized as (1) unemployed; (2) low (75% working time spent on sitting or standing, 25% activity); (3) moderate (25% sitting or standing, 75% activity with moderate intensity); (4) high (40% sitting or standing, 60% activity with high intensity) (58).

In the validation analysis of base-Q, 16 couples were randomly selected and invited to complete the same questionnaire twice over the study period with a time interval ranging from 15 days to 2 months. Intraclass correlation coefficients were higher than 0.60 (0.62–0.81), indicating moderate to good test-retest reliability (59).

### Covariates

Potential covariates include factors related to human fertility and IVF outcomes confirmed in previous studies (8, 9, 50, 54, 60). Information on maternal age (continuous), pre-pregnancy body mass index (BMI, continuous), educational level (middle school or below, high school, vocational or technical college, undergraduate, or postgraduate), the type (primary or secondary), cause (unexplained, male factor, female factor, or both) and duration (continuous) of infertility, COH protocol (long, short, antagonist, or others), insemination technique used (IVF, ICSI, or both), and the stage and quality of embryos transferred were extracted from the medical records. Infertility is the inability to conceive within 12 months of regular, unprotected sexual intercourse (1). In this study, the endpoint of infertility duration evaluation was the date of oocyte retrieval.

Other covariates including preconception psycho-mental status (stress, anxiety, and depression) and lifestyle factors (smoking, passive smoking, sleep quality, physical activity, and work intensity) were derived from the base-Q, as the first questionnaire completed before IVF treatment. Smoking status was categorized as (1) current smoker; (2) former smoker; (3) non-smoker. Passive smoking status was treated as a rank variable according to the frequency of exposure (< once a week; ≥once a week). Sleep quality and physical activity were treated as continuous variates. Work intensity was treated as a rank variable according to the categorization described above.

The confounders associated with the dietary pattern were also considered, such as the consumption of beverages (categorical), spicy food (categorical), and micronutrient supplement (continuous). In detail, due to non-quantitative data collection, the intake of beverages and spicy food were analyzed as rank variate and dichotomous variate (yes or no), respectively. The intakes of beverages were ranked based on the frequency (Never/Seldom; ≥once a month; < once a week; ≥once a week). Univariate associations of covariates with reproductive outcomes following IVF treatment are summarized in Supplementary Table 2.

### Statistical Analysis

Principal component analysis (PCA) with varimax rotation and k-means cluster analysis was used to extract dietary patterns (15). PCA is a data-driven technique that reduces the dimensions of the data and groups correlated variables to derive common components, in this case, dietary patterns (61). The number of factors reserved was based on the eigenvalue, factor interpretability, and the point at which the scree plot leveled off (Supplementary Figure 2). The coefficients of components describe the correlation between food groups and give interpretation to dietary patterns. We deemed those food items with coefficients ≥0.40 in each pattern to be important for the interpretability. The Bartlett test of sphericity and Kaiser-Mayer-Olkin test were used to test the applicability of analysis. Dietary pattern scores were assigned to reflect the adherence to each pattern for each participant. The scores were obtained by multiplying the coefficients by the corresponding standardized intake of each food group and summing it up. We divided the score of each pattern into quartiles (Q1-Q4) for further analysis and defined the lowest fourth as the reference.

K-means cluster analysis was performed on the percentages of weekly intake of the food groups, as described previously (25). We pre-specify the number of categories to be clustered, from 2 to 6, and determine the final number of mutually exclusive groups based on the actual meaning and interpretability of the results. In this way, all the participants were divided into different groups with an exclusive dietary pattern.

Descriptive statistics were used to describe the demographic, clinical, and lifestyle characteristics of each participant, shown as mean ± standard deviation (SD) for continuous variates or number (percentage) for categorical variates. Poisson regression with robust variance estimation was performed to explore the associations between the dietary patterns and the possibilities of normal fertilization, good-quality and transferable embryos formed on day 3, biochemical and clinical pregnancy, and live birth due to their high prevalence (62). Multivariable logistic regression was conducted to assess the effect of dietary patterns on the risks of early abortion, late abortion, preterm delivery, low birth weight, macrosomia, GDM, and hypertensive disorders during pregnancy. Tests for the linear trend were performed by using the median score in each quartile as a continuous variable. Only the covariates with a univariate association of *p* < 0.2 were adjusted in final models (57), as shown in Supplementary Table 2.

Given that maternal obesity has been widely reported to be associated with reproductive outcomes, including the success of IVF treatment, pregnancy complications, and even infant outcomes (63–65), we conducted stratified analyses based on pregnancy BMI categories (<25 kg/m^2^; ≥25 kg/m^2^) (29).

All statistical analyses were performed using SPSS version 22.0, and two-sided significance levels <0.05 were considered to be statistically significant.

## Results

### Demographic, Clinical, and Lifestyle Characteristics

Between April 2017 and April 2021, a total of 2,796 couples were recruited in our prospective cohort, among which, 1,500 couples were included in the analysis in this study after selection (Figure 1). The demographic and clinical characteristics of the study population are summarized in Table 1. The average age was 32.09 ± 4.33 years, and the average BMI was 23.35 ± 3.56 kg/m^2^. Most women were non-smoker (91.0%), and 54.9% of couples sought IVF treatment due to the female factor alone. There were 798 women (53.2%) who achieved biochemical pregnancy, 687 (45.8%) achieved clinical pregnancy, of which, 529 women (35.3%) had a live newborn, while 20 women were still at the stage of ongoing pregnancy.

**Table 1:** Demographic and clinical characteristics of the study population (*N* = 1,500).

**Characteristics**	**Data**
Maternal age (years)	32.09 ± 4.33
BMI (kg/m^2^)	23.35 ± 3.56
Educational level:
≤ middle school	332 (22.1%)
High school	223 (14.9%)
Vocational/technical college	296 (19.8%)
Undergraduate	554 (36.9%)
Postgraduate	95 (6.3%)
Infertility type:
Primary infertility	830 (55.3%)
Secondary infertility	570 (44.7%)
Duration of infertility (years)	3.81 ± 2.94
Infertility cause:
Unexplained	61 (4.1%)
Male factor	175 (11.7%)
Female factor	823 (54.8%)
Both	441 (29.4%)
COH protocol:
Long agonist	417 (27.8%)
Short agonist	31 (2.1%)
Antagonist	778 (51.9%)
Others	274 (18.2%)
Insemination method:
IVF	811 (54.1%)
ICSI	466 (31.0%)
IVF + ICSI	223 (14.9%)
Normally fertilized embryos	8.48 ± 5.52
Good-quality embryos on day 3	6.08 ± 4.39
Transferable embryos	4.28 ± 4.12
Stage of embryos transferred:
Cleavage	1,009 (67.3%)
Blastocyst	491 (32.7%)
Number of embryos transferred:
One	739 (49.3%)
Two	761 (50.7%)
Quality of embryos transferred:	
Good-quality	1,199 (79.9%)
Good-quality + non-good-quality	123 (8.2%)
Non-good-quality	178 (11.9%)
Biochemical pregnancy	798 (53.2%)
Clinical pregnancy	687 (45.8%)
Ectopic pregnancy	18 (2.3%)^a^
Early abortion (≤ 12 GWs)	88 (6.3%)
Late abortion (> 12 GWs)	31 (2.1%)^b^
Live birth	529 (35.3%)^b^
Preterm delivery	73 (4.9%)^b^
Low birth weight	51 (3.4%)^b^
Macrosomia	40 (2.7%)^b^
Gestational diabetes mellitus	90 (6.0%)^b^
Hypertensive disorders during pregnancy	37 (2.5%)^b^

*Data were described as mean ± SD or N (%)*.

a *The proportion was calculated as the number of cycles resulting in ectopic pregnancy divided by the number of cycles achieving biochemical pregnancy*.

b *20 women in a state of ongoing pregnancy were excluded from the analysis*.

The information on lifestyle, psycho-mental status, beverage and spicy food consumption, and micronutrient supplement intake of the study population is presented in Table 2. Most women had a moderate perceived stress scale score and good sleep quality, without anxiety or depression.

**Table 2:** Lifestyle, psycho-mental status, beverage and spicy food consumption, and micronutrient supplement intake of the study population (*N* = 1,500).

**Characteristics**	**Data**
Smoking status:	
Current smoker	31 (2.1%)
Former smoker	104 (6.9%)
Non-smoker	1,365(91.0%)
Passive smoking status:	
< once a week	1,076 (71.7%)
≥ once a week	424 (28.3%)
Perceived stress scale score:	
0–10	317 (21.1%)
11–20	1,050 (70.0%)
>20	133 (8.9%)
Anxiety status:	
No	1,396 (93.1%)
Mild	85 (5.6%)
Moderate	16 (1.1%)
Severe	3 (0.2%)
Depression status:	
Have symptoms of depression	475 (31.7%)
No symptoms of depression	1,025 (68.3%)
Sleep quality:	
Very good	855 (57.0%)
Fairly good	410 (27.4%)
Fairly bad	149 (9.9%)
Very bad	86 (5.7%)
Metabolic equivalents (min/week)	1,180.81 ± 2,174.11
Work intensity:	
Unemployed	988 (65.9%)
Low	491 (32.7%)
Moderate	17 (1.1%)
High	4 (0.3%)
Alcoholic beverages consumption	
Never/Seldom	1,454 (96.9%)
≥ Once a month; < once a week	39 (2.6%)
≥ Once a week	7 (0.5%)
Tea consumption	
Never/Seldom	1,261 (84.1%)
≥ Once a month; < once a week	118 (7.8%)
≥ Once a week; < everyday	75 (5.0%)
Everyday	46 (3.1%)
Coffee consumption	
Never/Seldom	1,367 (91.1%)
≥ Once a month; < once a week	63 (4.2%)
≥ Once a week; < everyday	36 (2.4%)
Everyday	34 (2.3%)
Functional beverages consumption	
Never/Seldom	1,458 (97.3%)
≥ once a month; < once a week	38 (2.5%)
≥ once a week; < everyday	2 (0.1%)
Everyday	2 (0.1%)
Spicy food intake:	
Yes	1,319 (88.0%)
No	181 (12.0%)
Folic acid supplement (mg/month)	4.80 ± 7.37
Multi-vitamins supplement (pill/week)	3.43 ± 4.97
Vitamin A supplement (mg/week)	0.35 ± 2.57
Vitamin B supplement (mg/week)	13.99 ± 96.45
Vitamin C supplement (mg/week)	76.75 ± 314.96
Vitamin D supplement (ug/week)	5.36 ± 25.23
Vitamin E supplement (mg/week)	2,294.57 ± 3,386.08
Calcium supplement (mg/week)	144.30 ± 724.25
Iron supplement (mg/week)	17.72 ± 249.46

*Data were described as mean ± SD or N (%)*.

### Dietary Patterns

**Figure 2:**
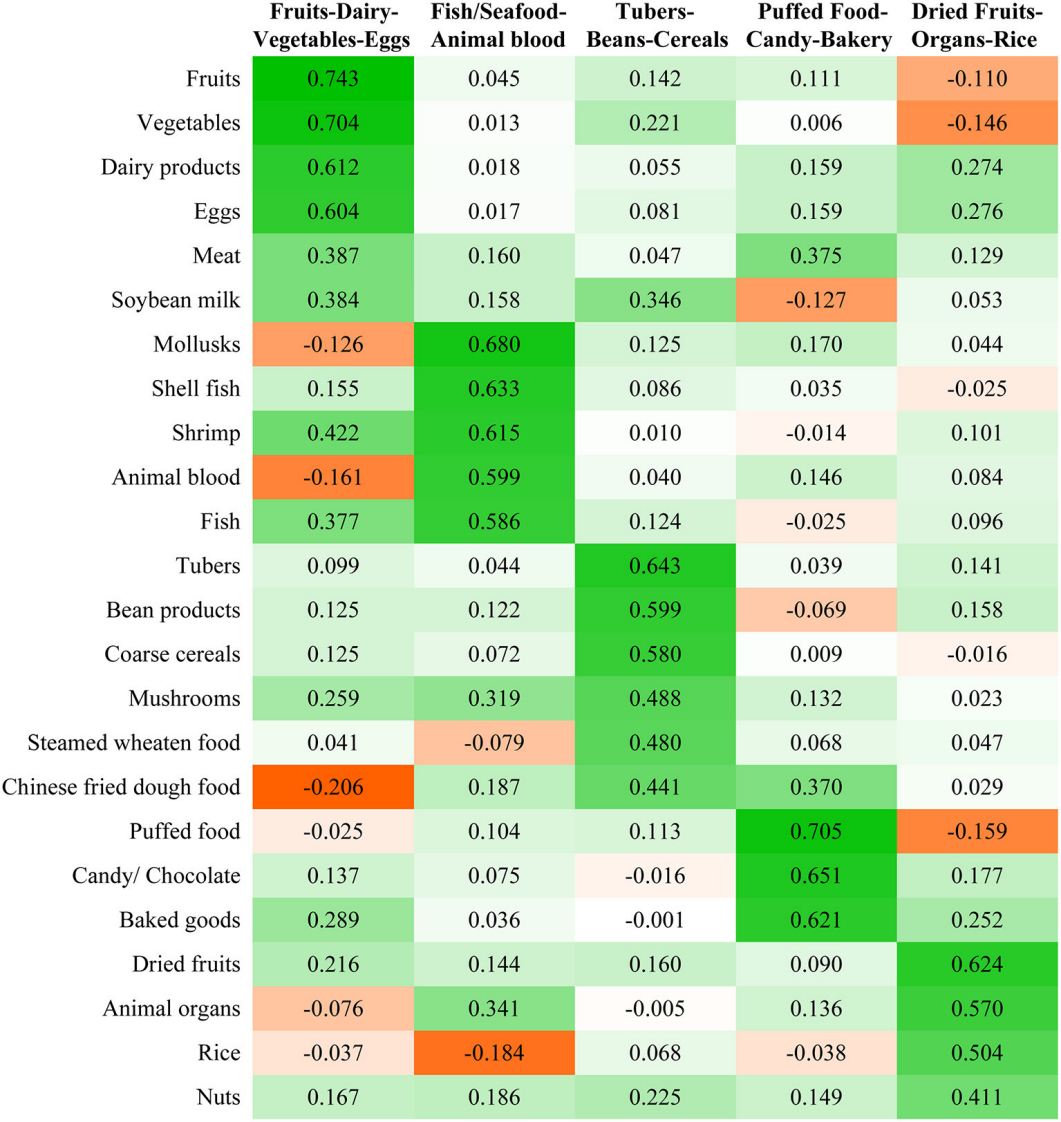
Factor loadings of food groups in each dietary pattern were identified using PCA. The color gradation denotes the strength and direction of the correlation between the food groups and dietary patterns. Deep green represents a relatively higher correlation (a higher intake) of the food groups with the corresponding dietary patterns. Deep orange represents relatively a lower correlation (a lower intake) of the food groups with the corresponding patterns. White represents no correlation between the food groups with the corresponding dietary pattern.

Five dietary patterns were identified by PCA (Figure 2) and accounted for 44.3% of the total variance in the diet. The first dietary pattern was characterized by frequent intakes of fruit, vegetables, dairy products, and eggs. The second dietary pattern had higher intakes of seafood (including mollusks, shellfish, and shrimp), fish, and animal blood. The third dietary pattern was characterized by more frequent intakes of tubers, bean products, and coarse cereals. The fourth dietary pattern had higher intakes of puffed food, candy/chocolate, and baked goods. The fifth dietary pattern was characterized by frequent intakes of dried fruits, animal organs, and rice. Each dietary pattern was named after the food groups with a factor loading >0.5, therefore, the five patterns were named as “Fruit-Dairy-Vegetables-Eggs,” “Fish/Seafood-Animal blood,” “Tubers-Beans-Cereals,” “Puffed food-Candy-Bakery,” and “Dried Fruits-Organs-Rice,” respectively.

Moreover, we identified four clusters of dietary patterns from the cluster analysis (Table 3). The first cluster was characterized by higher intakes of fruits and vegetables, named “Fruits-Vegetables.” The second cluster had a higher consumption of dairy products and was named “Dairy.” The third cluster was the most frequently consumed rice, and the fourth had relatively higher consumption of varied food groups, named “Rice” and “Varied,” respectively.

**Table 3:** Percentages (%) of weekly intake of 24 food groups assessed with a self-administered food frequency questionnaire across the four dietary patterns identified among 1,500 women in this prospective cohort.

**Food groups^a^**	**Dietary patterns**							
	**Fruits-Vegetables** **(*****n*** **=** **408)**		**Dairy (*****n*** **=** **280)**		**Rice (*****n*** **=** **232)**		**Varied (*****n*** **=** **580)**	
	**Mean**	**SD**	**Mean**	**SD**	**Mean**	**SD**	**Mean**	**SD**
Rice	10.09	7.08	8.86	5.95	34.17	10.97	11.49	6.18
Steamed wheaten foods	4.63	4.53	4.61	4.32	6.53	6.19	7.18	6.76
Coarse cereals	1.70	2.64	1.68	2.51	1.55	3.28	3.06	3.95
Chinese fried dough foods	0.41	0.92	0.51	1.22	0.73	1.89	0.97	1.59
Tubers	2.52	2.98	2.00	2.24	3.16	4.53	3.60	3.73
Bean products	2.11	2.69	2.21	2.76	2.24	2.51	3.58	3.90
Soybean milk	3.40	4.78	3.72	5.18	2.00	3.33	5.34	6.91
Mushrooms	1.82	2.29	1.54	1.67	1.31	1.66	2.39	2.23
Vegetables	18.82	9.46	9.70	5.07	12.61	8.35	10.11	5.64
Fruits	25.42	9.64	14.28	6.72	11.28	7.32	11.31	5.32
Dried fruits	1.20	2.04	1.32	2.11	0.93	2.62	1.80	2.68
Nuts	1.48	2.09	1.89	2.43	1.26	2.32	2.38	2.90
Animal organs	0.29	0.85	0.44	1.50	0.40	1.05	0.81	1.47
Animal blood	0.10	0.41	0.09	0.32	0.09	0.38	0.37	1.14
Shrimp	1.73	2.29	1.85	2.50	0.97	2.14	2.24	3.02
Fish	1.96	2.71	2.05	2.44	1.31	1.98	2.69	3.27
Mollusks	0.17	0.60	0.24	0.59	0.09	0.32	0.57	1.30
Shellfish	0.68	1.57	0.57	1.13	0.34	1.00	1.06	2.14
Eggs	5.73	4.46	6.59	4.25	5.21	4.79	6.72	5.41
Baked goods	2.52	3.42	3.89	4.18	2.01	3.26	4.04	4.62
Candy/Chocolate	0.97	1.88	1.33	2.69	0.94	1.68	1.98	2.94
Puffed food	0.51	1.48	0.64	1.34	0.57	1.95	1.16	2.23
Dairy	6.39	4.91	23.20	7.98	4.05	4.75	6.37	4.21
Meat	5.36	4.94	6.79	5.42	6.42	6.46	8.86	8.05

a *Percentage value (%) was calculated as the intake of the specific food group divided by total food intake*.

*The highest mean values are underlined and bolded*.

### Dietary Patterns and Reproductive Outcomes

Modified Poisson regression models were used to assess the associations between the dietary patterns derived by PCA and the outcomes of IVF treatment. In terms of embryo development, positive dose-response relationships were observed between “Fruit-Dairy-Vegetables-Eggs” score and normal fertilization and transferable embryos formed (*p* for trend = 0.034 and 0.003, respectively) (Table 4). Specifically, women in the highest vs. lowest quartile of “Fruit-Dairy-Vegetables-Eggs” score had increased likelihoods to gain normally fertilized oocytes (adjusted RR: 1.04, 95%CI: 1.01–1.08) and transferable embryos (adjusted RR: 1.09, 95%CI: 1.04–1.14). In the contrast, we observed that a higher “Tubers-Beans-Cereals” score was associated with a lower possibility of normal fertilization (adjusted RR: 0.95, 95%CI: 0.92–0.98) and the formation of transferable embryos (adjusted RR: 0.94, 95%CI: 0.90–0.98). Regarding pregnancy outcomes, we observed that women in the highest vs. lowest quartile of “Puffed food-Candy-Bakery” score had decreased probability to achieve biochemical pregnancy of 17% (95%CI:0.72–0.96) (Table 5). Moreover, the “Dried fruits-Organs-Rice” score was positively related to the likelihood to achieve biochemical pregnancy (adjusted RR: 1.20, 95%CI: 1.05–1.37 for Q3 vs. Q1). No significant relationship was observed between dietary patterns and the probability to gain good-quality embryos on day 3 and live birth (Tables 4, 5).

**Table 4:** Associations of the dietary patterns derived by principal component analysis with the likelihoods of normal fertilization, transferable embryos, and good-quality embryos on day 3.

**Dietary pattern**		**Normal fertilization^a^**			**Transferable embryos^b^**			**Good-quality embryos on day 3^c^**		
		**Adjusted RR**	**95% CI**	***P*_for trend_**	**Adjusted RR**	**95% CI**	***P*_for trend_**	**Adjusted RR**	**95% CI**	***P*_for trend_**
Fruits-Vegetables-Dairy-Eggs	Q1	Ref	–	0.034^*^	Ref	–	0.003^**^	Ref	–	0.150
	Q2	**1.07**	1.03, 1.10^**^		**1.08**	1.03, 1.13^**^		1.03	0.97, 1.10	
	Q3	1.02	0.99, 1.06		1.04	0.99, 1.09		1.04	0.97, 1.10	
	Q4	**1.04**	1.01, 1.08^*^		**1.09**	1.04, 1.14^**^		1.06	0.99, 1.13	
Fish/Seafood-Animal blood	Q1	Ref	–	0.526	Ref	–	0.355	Ref	–	0.689
	Q2	0.98	0.95, 1.01		0.97	0.92, 1.01		1.02	0.96, 1.09	
	Q3	1.00	0.97, 1.03		0.99	0.95, 1.04		0.99	0.93, 1.06	
	Q4	0.99	0.96, 1.02		0.97	0.93, 1.02		1.02	0.95, 1.08	
Tubers-Beans-Cereals	Q1	Ref	–	0.008^**^	Ref	–	0.006^**^	Ref	–	0.146
	Q2	0.98	0.95, 1.01		0.97	0.93, 1.01		1.00	0.94, 1.06	
	Q3	1.00	0.97, 1.03		**0.94**	0.90, 0.98^**^		**0.92**	0.87, 0.98^*^	
	Q4	**0.95**	0.92, 0.98^**^		**0.94**	0.90, 0.98^**^		0.96	0.90, 1.03	
Puffed food-Candy-Bakery	Q1	Ref	–	0.546	Ref	–	0.051	Ref	–	0.253
	Q2	0.98	0.95, 1.01		1.00	0.95, 1.04		1.02	0.96, 1.09	
	Q3	0.99	0.96, 1.02		0.96	0.91, 1.00		0.96	0.90, 1.02	
	Q4	0.99	0.96, 1.02		0.96	0.91, 1.00		0.97	0.91, 1.04	
Dried fruits-Organs-Rice	Q1	Ref	–	0.634	Ref	–	0.687	Ref	–	0.450
	Q2	0.97	0.94, 1.00		**1.06**	1.01, 1.11^*^		**1.08**	1.01, 1.14^*^	
	Q3	1.01	0.98, 1.04		**1.05**	1.00, 1.10^*^		1.01	0.95, 1.08	
	Q4	0.98	0.95, 1.02		1.00	0.96, 1.05		0.95	0.89, 1.02	

a *Models adjusted for age, BMI, the type and cause of infertility, insemination method, depression status, sleep quality, physical activity, alcoholic beverages consumption, spicy food intake, the supplement of vitamin D, calcium, and iron, and total energy*.

b *Models adjusted for age, BMI, educational level, the type, duration, and cause of infertility, the consumption of alcoholic beverages and tea, spicy food intake, the supplement of multi-vitamins, vitamin A, vitamin D, and calcium, and total energy*.

c *Models adjusted for age, educational level, the type, duration, and cause of infertility, physical activity, the consumption of tea and coffee, spicy food intake, the supplement of vitamin B, vitamin D, vitamin E, calcium, and iron, and total energy. The bold values indicate statistical significance*

* *P < 0.05*,

** *P < 0.01*.

**Table 5:** Associations of the dietary patterns derived by principal component analysis with biochemical pregnancy, clinical pregnancy, and live birth.

**Dietary pattern**		**Biochemical pregnancy^a^**			**Clinical pregnancy^a^**			**Live birth^b^**		
		**Adjusted RR**	**95% CI**	***P*_for trend_**	**Adjusted RR**	**95% CI**	***P*_for trend_**	**Adjusted RR**	**95% CI**	***P*_for trend_**
Fruits-Vegetables-Dairy-Eggs	Q1	Ref	–	0.817	Ref	–	0.381	Ref	–	0.298
	Q2	0.96	0.85, 1.10		0.90	0.78, 1.03		0.86	0.72, 1.03	
	Q3	1.01	0.89, 1.15		0.92	0.80, 1.07		0.84	0.70, 1.01	
	Q4	1.00	0.87, 1.15		0.91	0.78, 1.07		0.88	0.73, 1.06	
Fish/Seafood-Animal blood	Q1	Ref	–	0.267	Ref	–	0.478	Ref	–	0.568
	Q2	1.00	0.88, 1.14		1.01	0.86, 1.18		1.12	0.92, 1.35	
	Q3	0.91	0.79, 1.04		0.96	0.82, 1.12		1.07	0.89, 1.30	
	Q4	0.94	0.83, 1.07		0.96	0.82, 1.11		1.09	0.90, 1.32	
Tubers-Beans-Cereals	Q1	Ref	–	0.334	Ref	–	0.413	Ref	–	0.611
	Q2	1.02	0.89, 1.16		0.96	0.83, 1.12		0.96	0.80, 1.16	
	Q3	0.98	0.86, 1.12		0.94	0.81, 1.11		0.87	0.72, 1.06	
	Q4	1.07	0.94, 1.23		1.06	0.91, 1.23		1.04	0.87, 1.25	
Puffed food-Candy-Bakery	Q1	Ref	–	0.019^*^	Ref	–	0.113	Ref	–	0.053
	Q2	0.92	0.80, 1.04		0.90	0.77, 1.05		0.90	0.75, 1.09	
	Q3	0.98	0.86, 1.10		1.02	0.89, 1.18		1.00	0.83, 1.19	
	Q4	**0.83**	0.72, 0.96^*^		0.86	0.73, 1.01		**0.81**	0.67, 0.98^*^	
Dried fruits-Organs-Rice	Q1	Ref	–	0.048^*^	Ref	–	0.398	Ref	–	0.680
	Q2	**1.15**	1.00, 1.32^*^		1.12	0.96, 1.31		1.21	1.00, 1.47	
	Q3	**1.20**	1.05, 1.37^**^		**1.20**	1.03, 1.40^*^		**1.28**	1.06, 1.54^**^	
	Q4	1.15	1.00, 1.33		1.07	0.90, 1.26		1.06	0.87, 1.30	

a *Models adjusted for age, coffee consumption, the supplement of folic acid and vitamin D, total energy, and the stage and quality of embryos transferred*.

b *Models adjusted for age, infertility type, stress, coffee consumption, the supplement of folic acid and vitamin D, total energy, and the stage and quality of embryos transferred. The bold values indicate statistical significance*

* *P < 0.05*,

** *P < 0.01*.

Multivariate logistic regression models were conducted to explore the effects of preconception dietary patterns on neonatal outcomes and pregnancy complications. We detected that the women who more adhere to the “Fish/Seafood-Animal blood” pattern might have a lower risk for hypertensive disorders during pregnancy (adjusted OR: 0.37, 95%CI: 0.14–0.99 for Q3 vs. Q1) (Table 6). No significant association was observed between preconception dietary patterns and the risk of early abortion, late abortion, preterm delivery, low birth weight, macrosomia, and GDM (Table 6, Supplementary Tables 3, 4).

**Table 6:** Associations of the dietary patterns derived by principal component analysis with preterm delivery and hypertensive disorders during pregnancy (*N* = 1,480).

**Dietary pattern**		**Preterm delivery^a^**			**Hypertensive disorders during pregnancy^b^**		
		**Adjusted OR**	**95% CI**	***P*_for trend_**	**Adjusted OR**	**95% CI**	***P*_for trend_**
Fruits-Vegetables-Dairy-Eggs	Q1	Ref	–	0.921	Ref	–	0.276
	Q2	0.67	0.34, 1.30		0.93	0.34, 2.53	
	Q3	0.71	0.36, 1.40		1.62	0.63, 4.17	
	Q4	0.90	0.44, 1.88		1.64	0.56, 4.85	
Fish/Seafood-Animal blood	Q1	Ref	–	0.538	Ref	–	0.046^*^
	Q2	0.75	0.39, 1.41		0.62	0.25, 1.52	
	Q3	**0.32**	0.14, 0.71^**^		**0.37**	0.14, 0.99^*^	
	Q4	0.80	0.43, 1.49		0.45	0.17, 1.17	
Tubers-Beans-Cereals	Q1	Ref	–	0.556	Ref	–	0.689
	Q2	0.98	0.51, 1.88		1.86	0.71, 4.85	
	Q3	0.49	0.23, 1.08		1.23	0.44, 3.50	
	Q4	1.31	0.66, 2.59		1.51	0.49, 4.64	
Puffed food-Candy-Bakery	Q1	Ref	–	0.056	Ref	–	0.369
	Q2	0.82	0.43, 1.55		0.92	0.37, 2.27	
	Q3	0.57	0.29, 1.12		0.89	0.36, 2.16	
	Q4	0.55	0.26, 1.14		0.59	0.19, 1.82	
Dried fruits-Organs-Rice	Q1	Ref	–	0.382	Ref	–	0.648
	Q2	0.96	0.48, 1.93		1.01	0.38, 2.69	
	Q3	1.15	0.59, 2.26		1.89	0.77, 4.63	
	Q4	1.33	0.65, 2.74		1.05	0.34, 3.29	

a *Models adjusted for age, BMI, educational level, smoking status, depression status, functional beverages consumption, multi-vitamins supplement, and total energy*.

b *Models adjusted for age, BMI, type and cause of infertility, physical activity, the supplement of vitamin A and vitamin D, and total energy. The bold values indicate statistical significance*

* *P < 0.05*,

** *P < 0.01*.

Furthermore, we explore the relationship between the clusters of dietary patterns and reproductive outcomes taking “Fruits-Vegetables” as the reference group (Table 7). In modified Poisson regression models, we observed that women in the “Dairy” cluster had a higher possibility of the formation of transferable embryos (adjusted RR: 1.08, 95%CI: 1.03–1.13) and good-quality embryos on day 3 (adjusted RR: 1.07, 95%CI: 1.00–1.15) compared to women in the “Fruits-Vegetables” cluster. Moreover, in multivariate logistic regression models, women with a higher intake of rice were at a higher risk of delivering macrosomia (adjusted OR: 2.74, 95%CI: 1.02–7.35).

**Table 7:** Associations between the dietary patterns derived by cluster analysis and reproductive outcomes.

**Reproductive outcomes**	**Dietary pattern**			
	**Fruits-Vegetables**	**Dairy**	**Rice**	**Varied**
Normal fertilization^a^	Ref	1.03 (0.99, 1.06)	1.02 (0.99, 1.06)	1.01 (0.98, 1.03)
Transferable embryos^b^	Ref	1.08 (1.03, 1.13)^**^	1.03 (0.98, 1.09)	0.98 (0.94, 1.02)
Good-quality embryos on day 3^c^	Ref	1.07 (1.00, 1.15)^*^	1.05 (0.98, 1.13)	1.00 (0.94, 1.06)
Biochemical pregnancy^d^	Ref	0.93 (0.80, 1.08)	1.07 (0.93, 1.23)	0.98 (0.87, 1.10)
Clinical pregnancy^d^	Ref	0.87 (0.73, 1.04)	1.09 (0.92, 1.28)	1.04 (0.91, 1.19)
Live birth^e^	Ref	0.79 (0.63, 1.01)	1.04 (0.84, 1.28)	1.05 (0.89, 1.23)
Early abortion^f^	Ref	1.76 (0.93, 3.34)	1.50 (0.75, 2.99)	1.05 (0.58, 1.92)
Late abortion^g^^,^^m^	Ref	0.35 (0.09, 1.34)	0.79 (0.24, 2.62)	0.94 (0.40, 2.18)
Preterm delivery^h^^,^^m^	Ref	1.10 (0.47, 2.56)	1.94 (0.91, 4.11)	1.80 (0.93, 3.46)
Low birth weight^i^^,^^m^	Ref	0.66 (0.24, 1.78)	1.54 (0.67, 3.55)	1.17 (0.57, 2.40)
Macrosomia^j^^,^^m^	Ref	0.89 (0.25, 3.12)	2.74 (1.02, 7.35)^*^	1.91 (0.78, 4.69)
Gestational diabetes mellitus^k^^,^^m^	Ref	0.45 (0.20, 1.01)	0.64 (0.30, 1.36)	1.25 (0.74, 2.10)
Hypertensive disorders during pregnancy^l^^,^^m^	Ref	0.92 (0.34, 2.47)	0.66 (0.22, 1.96)	0.79 (0.34, 1.82)

a *Models adjusted for age, BMI, the type and cause of infertility, insemination method, depression status, sleep quality, physical activity, alcoholic beverages consumption, spicy food intake, the supplement of vitamin D, calcium, and iron, and total energy*.

b *Models adjusted for age, BMI, educational level, the type, duration, and cause of infertility, the consumption of alcoholic beverages and tea, spicy food intake, the supplement of multi-vitamins, vitamin A, vitamin D, and calcium, and total energy*.

c *Models adjusted for age, educational level, the type, duration, and cause of infertility, physical activity, the consumption of tea and coffee, spicy food intake, the supplement of vitamin B, vitamin D, vitamin E, calcium, and iron, and total energy*.

d *Models adjusted for age, coffee consumption, the supplement of folic acid and vitamin D, total energy, and the stage and quality of embryos transferred*.

e *Models adjusted for age, infertility type, stress, coffee consumption, the supplement of folic acid and vitamin D, total energy, and the stage and quality of embryos transferred*.

f *Models adjusted for BMI, anxiety status, the supplement of vitamin D and vitamin E, and total energy*.

g *Models adjusted for BMI, cause, stress, depression, vitamin C supplement, and total energy*.

h *Models adjusted for age, BMI, educational level, smoking status, depression status, functional beverages consumption, multi-vitamins supplement, and total energy*.

i *Models adjusted for age, depression status, the supplement of multi-vitamin and vitamin B, and total energy*.

j *Models adjusted for age, BMI, educational level, stress, physical activity, work intensity, the supplement of folic acid, vitamin A, and vitamin D, and total energy*.

k *Models adjusted for age, BMI, smoking status, stress, sleep quality, the consumption of alcoholic and functional beverages, spicy food intake, vitamin E supplement, and total energy*.

l *Models adjusted for age, BMI, type and cause of infertility, physical activity, the supplement of vitamin A and vitamin D, and total energy*.

m *20 women in a state of ongoing pregnancy were excluded from the analysis. The bold values indicate statistical significance*

* *P < 0.05*,

** *P < 0.01*.

The results of stratified analyses revealed that the positive relationship between the “Fruit-Dairy-Vegetables-Eggs” score and normal fertilization and the inverse association of the “Fish/Seafood-Animal blood” score with hypertensive disorders during pregnancy were exhibited only among women with BMI ≥25 kg/m^2^ (Supplementary Table 5). The associations between preconception diet and other reproductive outcomes were consistent across maternal pre-pregnancy BMI categories (data not shown).

## Discussion

In the present study, we used PCA to identify preconception dietary patterns in an ongoing prospective cohort, and then assessed the association between dietary patterns and reproductive outcomes. In terms of IVF intermediate and clinical outcomes, we detected that the women who are more inclined to the “Fruits-Vegetables-Dairy-Eggs” pattern and less adherent to the “Tubers-Beans-Cereals” pattern might be more likely to achieve normally fertilized and transferable embryos. In addition, we observed that a lower “Puffed food-Candy-Bakery” score and higher “Dried fruits-Organs-Rice” score were related to a higher likelihood to achieve biochemical pregnancy. We further clustered the dietary patterns based on the proportion of food groups consumed and found that frequent dairy products consumption was beneficial to embryo quality while frequent rice consumption was associated with a higher risk of macrosomia compared to the “Fruits-Vegetables” cluster. However, no association between dietary patterns and clinical pregnancy or live birth was observed.

The MedDiet, which refers to the dietary pattern of European countries on the Mediterranean coast, is characterized by a high intake of cereals, legumes, fruits, nuts, vegetables, fish, and olive oil while a low intake of dairy products, meat, poultry, saturated lipids, and a regularly moderate intake of alcohol (33). Most previous studies exploring the impact of pre-treatment dietary patterns on IVF success focused on compliance to the MedDiet but reached different conclusions (29, 33–35). A study conducted on 244 non-obese Greece women revealed that a beneficial 5-point increase in the MedDietScore associates with ~2.7 times higher likelihood of achieving clinical pregnancy and live birth among women <35 years (29). Sun et al. (33) performed a prospective study among 590 Chinese women and suggested that higher adherence to MedDiet may improve embryo yield, while not increasing IVF success rate (33). In contrast, no clear association was observed between MedDiet scores and IVF success in an Italian cohort study (35). Furthermore, the prospective Environment and Reproductive Health (EARTH) study, which evaluated the correlation between preconception dietary patterns and IVF outcomes, concluded that commonly recommended dietary advice such as adhering to the MedDiet may not provide the most appropriate guidance for women undergoing infertility treatment (34). Therefore, individual assessment of dietary patterns is of great significance for clinical practice.

To date, only two studies investigated the association between dietary patterns and IVF outcomes identifying individual patterns based on specific populations (28, 32). Vujkovic et al. (28) identified two dietary patterns namely “health conscious-low processed” and “Mediterranean” dietary patterns among 161 Dutch women (28). They revealed that a preconception “Mediterranean” diet, characterized by high intakes of vegetable oil, fish, legumes, and vegetables but low intakes of snacks, contributes to the success of achieving biochemical pregnancy (OR:1.4, 95%CI:1.0–1.9), which may in part support our finding of the inverse relationship between “Puffed food-Candy-Bakery” diet and biochemical pregnancy (adjusted RR:0.83, 95%CI:0.72–0.96). Interestingly, as reported by Vujkovic et al. (28), preconception “Mediterranean” diet could contribute to increases in blood folate concentration and vitamin B6 levels in both blood and follicular fluid. Folate and vitamin B6 have been suggested to be beneficial to oocyte quality and embryonic development (66–68). However, no significant association of dietary patterns with fertilization rate and embryo quality was detected by Vujkovic et al. (28), which may be attributed to the limited sample size and the absence of adjustment for confounders associating diet and lifestyle. The Rotterdam Periconceptional Cohort (Predict) Study recruited 135 women with spontaneous pregnancy and 93 women who achieved pregnancies upon IVF/ICSI treatment. Three dietary patterns were identified in the Predict Study, “High vegetables, fruits and grain,” “High solid fat, snacks and sugars,” and “High fish and olive oil, low meat,” while no significant association between periconceptional maternal dietary pattern and first-trimester embryonic growth was observed in the IVF/ICSI subgroup (32). Besides limited sample sizes and differences in the statistical method used, the discordance between the results of these two studies and ours may also be attributed to differences in demographic characteristics and long-term dietary culture of western and eastern.

An epidemical study conducted among 644 women seeking infertility treatment in an agricultural region suggested that drinking three or more glasses of milk per day was negatively related to the risk of female infertility (OR: 0.3, 95%CI: 0.1–0.7) (69), which may partly support our findings of the beneficial effect of dairy intake on embryological parameters. However, the study on the correlation between maternal dairy intake and infertility treatment outcomes was limited. The Environment and Reproductive Health (EARTH) study by Afeiche et al. (70) reported that total dairy food consumption was positively correlated with live birth among women ≥35 years of age, while it was not related to ovarian response, embryological, or clinical pregnancy outcomes (70). Notably, the EARTH study recruited the women with multiple IVF cycles, on the contrary, only the women undergoing their first IVF cycle were included in the present study to avoid introducing bias, which may be a potential reason for inconsistent results.

Regarding pregnancy complications, we observed that the women who were more adherent to the “Fish/Seafood-Animal blood” pattern might have a lower risk for hypertensive disorders during pregnancy (adjusted OR: 0.37, 95%CI: 0.14–0.99). Several studies explored the effects of dietary patterns on pregnancy complications (19, 71, 72), however, to the best of our knowledge, there is a lack of study conducted on the IVF population with the same aim. Jarman et al. (19) explored the relationship of pre-pregnancy diet with pregnancy complications based on an ongoing prospective cohort in Canada and suggested that higher “healthy” pattern scores were associated with lower odds of developing gestational hypertension during pregnancy (adjusted OR: 0.6, 95% CI: 0.4–0.9) (19). In detail, the “healthy” pattern in Jarman et al.'s study referred to frequent intakes of vegetables, fruit, oils, brown pasta or rice, fish, tomatoes, and white pasta. Another longitudinal study conducted on the Danish National Birth cohort revealed a protective association of seafood diet with gestational hypertension (OR:0.86, 95% CI: 0.77–0.95) and pre-eclampsia (OR: 0.79, 95% CI: 0.65–0.97) (71). The results of these two studies were in accordance with ours, and hence remind us of the potentially important role of pre-conception fish/seafood intake in preventing hypertensive disorders during pregnancy. In addition, we detected that the women with frequent consumption of rice were at a higher risk of delivering macrosomia (adjusted OR: 2.74, 95%CI: 1.02–7.35) in this study. A study conducted by (72) based on the Norwegian Mother and Child Cohort Study included 65,904 pregnant women to explore the effect of dietary patterns in pregnancy on birth weight (72). Taking the high Western group with the highest intake of carbohydrates as the reference, (72) revealed a lower birth weight in the high prudent group with the lowest intake of carbohydrates. Combined with our findings, at any stage of pregnancy, from preconception to gestational period, excessive consumption of high glycaemic index carbohydrates may be detrimental. However, there is a lack of study assessing the correlation between the pre-pregnancy diet and the risk of macrosomia, hence, more studies are warranted to verify our results.

In stratified analyses, we detected that the effects of the preconception diet on fertilization and the risk of hypertensive disorders during pregnancy were not consistent across BMI categories. Previous studies have reported that BMI above the normal range was negatively associated with IVF outcomes (63, 73, 74). Therefore, we assumed that the protective effect of a healthier diet at the pre-treatment stage may be more significant for overweight and obese women.

This is the first study to explore the effects of preconception dietary patterns on IVF outcomes ranging from embryo development to pregnancy complications in a Chinese population. Given the dramatic differences between western and eastern eating habits, a study using individual assessment of dietary patterns was necessary. In addition, several lifestyle factors including psycho-mental status, physical activity, and sleep quality were evaluated in this study using validated questionnaires and were adjusted in the final analyses, which might improve the accuracy and credibility of our results. Our findings revealed associations of preconception diet with reproductive outcomes following IVF treatment, including fertilization, embryo quality, and pregnancy complications, which may provide important clues for dietary guidance during preconception counseling. Moreover, our results preliminarily indicate that diet prior to pregnancy may be an important target for interventions to achieve a better reproductive outcome.

Inherent to the observational design of the present study, several limitations have to be acknowledged. First, the comparison with results from other studies is challenging due to the observational, data-driven approach of deriving dietary patterns. Although the FFQ used in this study was derived from a validated Chinese dietary FFQ and previous studies have suggested that it can be used to classify the subjects according to their food consumption in 1 year, there is still a potential measurement bias due to retrospective assessment, which may lead to misclassification. Moreover, 24 non-overlapping food groups classified in this study may be limited to cover habitual dietary intake. As for the statistical analyses, numerous statistical tests were conducted that may cause type-I error inflation and generate false-positive results. Notably, although significant, the effect sizes for fertilization and embryo quality were relatively small, therefore, the results are preliminary and their clinical relevance should be interpreted carefully. Furthermore, 41.4% of the male partner in this study had reproductive disorders, while their pre-treatment diets and lifestyles were not adjusted or discussed due to the limitation of data, hence, a more comprehensive study considering both male and female pre-treatment diets is warranted. In addition, the participants recruited in our ongoing prospective cohort were all from north-eastern China, thus a national, multicenter, large-sample-size study should be conducted to verify our findings. Finally, blood metabolomics testing might be conducive to obtaining a more accurate conclusion, and further studies to explore underlying mechanisms were necessary.

## Data Availability Statement

The original contributions presented in the study are included in the article/Supplementary Material, further inquiries can be directed to the corresponding authors.

## Author Contributions

JT and SW: conceptualization. SW: methodology and writing–original draft preparation. SW and XZhan: formal analysis. XZhao, XH, SZ, and PL: investigation. JT: resources and funding acquisition. PL: data curation. All authors contributed to the article and approved the submitted version.

## Funding

This research was funded by National Key Research and Development Program (2018YFC1004203), Major Special Construction Plan for Discipline Construction Project of China Medical University (3110118033), Shengjing Freelance Researcher Plan of Shengjing Hospital of China Medical University, National Natural Science Foundation of China (82071601/61873257), Key Research and Development Program of Liaoning Province (2018020222), and Central Government Special Fund for Local Science and Technology Development (2020JH6/10500006).

## Conflict of Interest

The authors declare that the research was conducted in the absence of any commercial or financial relationships that could be construed as a potential conflict of interest.

## Publisher's Note

All claims expressed in this article are solely those of the authors and do not necessarily represent those of their affiliated organizations, or those of the publisher, the editors and the reviewers. Any product that may be evaluated in this article, or claim that may be made by its manufacturer, is not guaranteed or endorsed by the publisher.
