# Supplementary material for: Preconception Dietary Patterns and Associations With IVF Outcomes: An Ongoing Prospective Cohort Study
Source: Front Nutr. 2022 Feb 16;9:808355. doi: 10.3389/fnut.2022.808355 (PMC8888455; doi:10.3389/fnut.2022.808355)
Supplement: Supplementary file 1 [file Data_Sheet_1.docx]

Supplementary Material

# Supplementary Tables

**Supplementary Table 1.** Food group list and beverage kinds in the food frequency questionnaire (FFQ).

| Classification | Groups |
| --- | --- |
| Food | Rice |
|  | Steamed wheaten foods (steamed /stuffed buns, noodles, etc.) |
|  | Coarse cereals (wheat, corn, sorghum, oats, etc.) |
|  | Chinese fried dough foods (deep-fried dough sticks etc.) |
|  | Tubers (potato, sweet potato, taro, Chinese yam, etc.) |
|  | Bean products (tofu, dried bean curd, sliced bean curd, etc.) |
|  | Soybean milk |
|  | Mushrooms |
|  | Vegetables (leafy vegetables, cauliflower, carrots, broccoli, etc.) |
|  | Fruits (cherry, grapefruit, orange, watermelon, etc.) |
|  | Dried fruit (red dates, longan, etc.) |
|  | Nuts (peanut, walnut, hazelnut, etc.) |
|  | Animal organs (animal liver/heart/kidney etc.) |
|  | Animal blood |
|  | Shrimp |
|  | Fish |
|  | Mollusks (squid, jellyfish, etc.) |
|  | Shellfish |
|  | Egg |
|  | Baked goods (bread, biscuits, cake, etc.) |
|  | Candy/ Chocolate |
|  | Puffed food |
|  | Dairy (fresh milk, yogurt, milk powder, etc.) |
|  | Meat (pork, beef, lamb, poultry, etc.) |
| Beverage | Alcoholic beverages |
|  | Coffee |
|  | Tea |
|  | Functional beverages |

**Supplementary Table 2.** Univariate association with IVF treatment outcomes (*p* value).

|  | Normal fertilization | Transferable embryos | Good-quality embryos | Biochemical pregnancy | Clinical  pregnancy | Live birth | Early abortion | Late abortion | Preterm delivery | Low birth weight | Macroso-mia | GDM | Hypertensive disorders during pregnancy |
| --- | --- | --- | --- | --- | --- | --- | --- | --- | --- | --- | --- | --- | --- |
| Age | **<0.001**** | **0.009**** | **0.003**** | **<0.001**** | **<0.001**** | **<0.001**** | 0.423 | 0.658 | **0.004**** | **0.018*** | **0.013*** | **0.034*** | **0.144^#^** |
| BMI | **<0.001**** | **0.079^#^** | 0.487 | 0.274 | 0.331 | 0.403 | **0.038*** | **0.024*** | **0.028*** | 0.266 | **0.034*** | **<0.001**** | **<0.001**** |
| educational level | 0.593 | **0.026*** | **<0.001**** | 0.965 | 0.920 | 0.768 | 0.221 | 0.679 | **0.112^#^** | 0.494 | **0.102^#^** | 0.375 | 0.401 |
| Infertility type | **0.005**** | **0.031*** | **0.151^#^** | 0.440 | 0.846 | **0.150^#^** | 0.209 | 0.674 | 0.563 | 0.949 | 0.357 | 0.485 | **0.138^#^** |
| Infertility duration | 0.368 | **0.061^#^** | **0.046*** | 0.226 | 0.462 | 0.232 | 0.561 | 0.733 | 0.643 | 0.672 | 0.800 | 0.624 | 0.743 |
| Infertility cause | **<0.001**** | **0.027*** | **0.045*** | 0.611 | 0.219 | 0.534 | 0.860 | **0.050^#^** | 0.848 | 0.739 | 0.876 | 0.270 | **0.146^#^** |
| COH protocol | 0.297 | 0.372 | 0.388 | 0.281 | 0.414 | 0.290 | 0.856 | 0.833 | 0.495 | 0.708 | 0.535 | 0.212 | 0.832 |
| insemination technique | **<0.001**** | 0.441 | 0.500 | 0.301 | 0.420 | 0.280 | 0.271 | 0.286 | 0.735 | 0.519 | 0.986 | 0.790 | 0.693 |
| Smoking status | 0.987 | 0.600 | 0.409 | 0.654 | 0.675 | 0.725 | 0.277 | 0.785 | **0.006**** | 0.375 | 0.505 | **0.045*** | 0.402 |
| Passive smoking status | 0.200 | 0.366 | 0.631 | 0.526 | 0.926 | 0.688 | 0.937 | 0.598 | 0.510 | 0.466 | 0.525 | 0.853 | 0.817 |
| Perceived stress scale score | 0.565 | 0.882 | 0.262 | 0.293 | 0.368 | **0.054^#^** | 0.644 | **0.151^#^** | 0.266 | 0.549 | **0.075^#^** | **0.066^#^** | 0.633 |
| Anxiety status | 0.695 | 0.454 | 0.264 | 0.753 | 0.679 | 0.222 | **0.072^#^** | 0.737 | 0.510 | 0.335 | 0.512 | 0.271 | 0.312 |
| Depression status | **0.025*** | 0.675 | 0.505 | 0.200 | 0.204 | 0.487 | 0.990 | **0.141^#^** | **0.184^#^** | **0.116^#^** | 0.655 | 0.740 | 0.532 |
| Sleep quality | **0.167^#^** | 0.819 | 0.976 | 0.603 | 0.467 | 0.765 | 0.397 | 0.292 | 0.844 | 0.322 | 0.586 | **0.157^#^** | 0.713 |
| Physical activity | **0.021*** | 0.438 | **0.020*** | 0.397 | 0.315 | 0.851 | 0.781 | 0.954 | 0.390 | 0.515 | **0.043*** | 0.336 | **0.165^#^** |
| Work intensity | 0.429 | 0.932 | 0.285 | 0.592 | 0.611 | 0.431 | 0.662 | 0.246 | 0.661 | 0.351 | **0.057** | 0.495 | 0.917 |
| Alcoholic beverages  consumption | **0.157^#^** | **0.006**** | 0.285 | 0.451 | 0.946 | 0.450 | 0.415 | 0.685 | 0.774 | 0.953 | 0.559 | **0.086^#^** | 0.818 |
| Tea consumption | 0.303 | **0.060^#^** | **0.001**** | 0.609 | 0.819 | 0.820 | 0.777 | 0.346 | 0.963 | 0.330 | 0.676 | 0.464 | 0.242 |
| Coffee consumption | 0.224 | 0.911 | **0.031*** | **0.080^#^** | **0.085^#^** | **0.125^#^** | 0.823 | 0.969 | 0.458 | 0.447 | 0.444 | 0.872 | 0.216 |
| Functional beverages | 0.386 | 0.384 | 0.349 | 0.893 | 0.591 | 0.267 | 0.338 | 0.996 | **0.047*** | 0.653 | 0.587 | **0.004**** | 0.999 |
| Spicy food intake | **<0.001**** | **0.017*** | **0.002**** | 0.548 | 0.506 | 0.689 | 0.415 | 0.685 | 0.774 | 0.953 | 0.559 | **0.086^#^** | 0.818 |
| Folic acid  supplement | 0.985 | 0.501 | 0.662 | **0.025*** | **0.003**** | **0.023*** | 0.377 | 0.391 | 0.944 | 0.670 | **0.033** | 0.548 | 0.355 |
| Multi-vitamins  supplement | 0.390 | **0.173^#^** | 0.881 | 0.919 | 0.560 | 0.337 | 0.942 | 0.771 | **0.153^#^** | **0.109^#^** | 0.498 | 0.533 | 0.467 |
| Vitamin A  supplement | 0.784 | **0.117^#^** | 0.363 | 0.508 | 0.713 | 0.955 | 0.918 | 0.996 | 0.412 | 0.468 | **0.053^#^** | 0.357 | **0.042*** |
| Vitamin B  supplement | 0.668 | 0.431 | **0.059^#^** | 0.252 | 0.386 | 0.425 | 0.942 | 0.520 | 0.372 | **0.148^#^** | 0.621 | 0.280 | 0.561 |
| Vitamin C  supplement | 0.227 | 0.358 | 0.224 | 0.854 | 0.984 | 0.700 | 0.724 | **0.138^#^** | 0.361 | 0.934 | 0.839 | 0.320 | 0.497 |
| Vitamin D  supplement | **0.070*** | **0.016*** | **<0.001**** | **0.016*** | **0.001**** | **0.009**** | **0.188^#^** | 0.398 | 0.785 | 0.333 | **0.029*** | 0.928 | **0.085^#^** |
| Vitamin E  supplement | 0.928 | 0.722 | **0.035*** | 0.868 | 0.775 | 0.473 | **0.103^#^** | 0.516 | 0.814 | 0.682 | 0.639 | **0.144^#^** | 0.576 |
| Calcium supplement | **0.133^#^** | **0.001**** | **<0.001**** | 0.764 | 0.599 | 0.470 | 0.246 | 0.562 | 0.313 | 0.743 | 0.766 | 0.500 | 0.788 |
| Iron supplement | **0.003**** | 0.344 | **0.049*** | 0.334 | 0.412 | 0.613 | 0.621 | 0.990 | 0.532 | 0.989 | 0.850 | 0.487 | 0.980 |
| Total energy | **0.124^#^** | **0.147^#^** | **0.141^#^** | **0.136^#^** | **0.137^#^** | **0.144^#^** | **0.150^#^** | **0.031*** | **0.175^#^** | **0.187^#^** | **0.178^#^** | **0.183^#^** | **0.152^#^** |
| Stage of embryo transferred | **-** | **-** | **-** | **<0.001**** | **<0.001**** | **<0.001**** | **-** | **-** | **-** | **-** | **-** | **-** | **-** |
| The number of embryo transferred | - | - | - | 0.512 | 0.676 | 0.642 | **-** | **-** | **-** | **-** | **-** | **-** | **-** |
| Quality of embryo transferred | - | - | - | **<0.001**** | **<0.001**** | **<0.001**** | **-** | **-** | **-** | **-** | **-** | **-** | **-** |

**Supplementary Table 3.** Associations of the dietary patterns derived by principal component analysis with early abortion and late abortion.

| Dietary pattern | | Early abortion ^a^ | | |  | Late abortion ^b^ | | |
| --- | --- | --- | --- | --- | --- | --- | --- | --- |
|  |  | Adjusted OR | 95% CI | *P* _for trend_ |  | Adjusted OR | 95% CI | *P* _for trend_ |
| Fruits-Vegetables-Dairy-Eggs | Q1 | Ref | - | 0.725 |  | Ref | - | 0.968 |
|  | Q2 | 0.75 | 0.39, 1.45 |  |  | 1.38 | 0.46, 4.10 |  |
|  | Q3 | 1.17 | 0.64, 2.12 |  |  | 1.04 | 0.34, 3.18 |  |
|  | Q4 | 1.02 | 0.53, 1.96 |  |  | 1.14 | 0.38, 3.39 |  |
| Fish/Seafood-Animal blood | Q1 | Ref | - | 0.059 |  | Ref | - | 0.320 |
|  | Q2 | 0.80 | 0.44, 1.44 |  |  | **0.25** | **0.07, 0.89*** |  |
|  | Q3 | 0.61 | 0.32, 1.14 |  |  | **0.31** | **0.10, 0.99*** |  |
|  | Q4 | 0.55 | 0.30, 1.02 |  |  | 0.67 | 0.29, 1.56 |  |
| Tubers-Beans-Cereals | Q1 | Ref | - | 0.947 |  | Ref | - | 0.554 |
|  | Q2 | 1.08 | 0.57, 2.03 |  |  | 1.11 | 0.35, 3.53 |  |
|  | Q3 | 1.22 | 0.66, 2.26 |  |  | 1.43 | 0.49, 4.12 |  |
|  | Q4 | 1.02 | 0.53, 1.97 |  |  | 1.35 | 0.47, 3.84 |  |
| Puffed food-Candy-Bakery | Q1 | Ref | - | 0.360 |  | Ref | - | 0.721 |
|  | Q2 | 0.92 | 0.46, 1.85 |  |  | 0.80 | 0.28, 2.32 |  |
|  | Q3 | 1.56 | 0.84, 2.88 |  |  | 0.62 | 0.20, 1.87 |  |
|  | Q4 | 1.28 | 0.66, 2.49 |  |  | 1.16 | 0.43, 3.08 |  |
| Dried fruits-Organs-Rice | Q1 | Ref | - | 0.403 |  | Ref | - | 0.497 |
|  | Q2 | 0.82 | 0.43, 1.58 |  |  | 0.83 | 0.30, 2.27 |  |
|  | Q3 | 1.09 | 0.59, 1.99 |  |  | 0.78 | 0.28, 2.15 |  |
|  | Q4 | 1.24 | 0.65, 2.35 |  |  | 0.69 | 0.24, 2.00 |  |

^a^ Models adjusted for BMI, anxiety status, the supplement of vitamin D, and vitamin E, and total energy.

^b^ Models adjusted for BMI, cause, stress, depression, vitamin C supplement, and total energy. 20 women in a state of ongoing pregnancy were excluded from the analysis

**Supplementary Table 4.** Associations of the dietary patterns derived by principal component analysis with low birth weight, macrosomia, and gestational diabetes mellitus (N = 1480).

| Dietary pattern | | Low birth weight ^a^ | | |  | Macrosomia ^b^ | | |  | Gestational diabetes mellitus ^c^ | | |
| --- | --- | --- | --- | --- | --- | --- | --- | --- | --- | --- | --- | --- |
|  |  | Adjusted OR | 95% CI | *P* _for trend_ |  | Adjusted OR | 95% CI | *P* _for trend_ |  | Adjusted OR | 95% CI | *P* _for trend_ |
| Fruits-Vegetables-Dairy-Eggs | Q1 | Ref | - | 0.169 |  | Ref | - | 0.385 |  | Ref | - | 0.597 |
|  | Q2 | 0.57 | 0.26, 1.23 |  |  | 0.70 | 0.31, 1.61 |  |  | 0.83 | 0.45, 1.54 |  |
|  | Q3 | 0.56 | 0.26, 1.21 |  |  | 0.55 | 0.21, 1.41 |  |  | 0.73 | 0.38, 1.38 |  |
|  | Q4 | 0.59 | 0.25, 1.40 |  |  | 0.65 | 0.23, 1.83 |  |  | 1.15 | 0.60, 2.19 |  |
| Fish/Seafood-Animal blood | Q1 | Ref | - | 0.259 |  | Ref | - | 0.070 |  | Ref | - | 0.744 |
|  | Q2 | 1.05 | 0.51, 2.17 |  |  | **3.69** | **1.40, 9.71**** |  |  | 1.12 | 0.59, 2.12 |  |
|  | Q3 | **0.22** | **0.07, 0.69**** |  |  | 1.50 | 0.52, 4.37 |  |  | 0.94 | 0.49, 1.81 |  |
|  | Q4 | 0.93 | 0.44, 1.94 |  |  | 0.55 | 0.15, 2.08 |  |  | 1.13 | 0.62, 2.08 |  |
| Tubers-Beans-Cereals | Q1 | Ref | - | 0.194 |  | Ref | - | 0.392 |  | Ref | - | 0.705 |
|  | Q2 | 0.82 | 0.40, 1.69 |  |  | 1.31 | 0.57, 3.04 |  |  | 1.00 | 0.53, 1.87 |  |
|  | Q3 | **0.28** | **0.10, 0.78*** |  |  | 0.70 | 0.27, 1.83 |  |  | 0.88 | 0.47, 1.66 |  |
|  | Q4 | 0.80 | 0.37, 1.73 |  |  | 0.75 | 0.27, 2.12 |  |  | 1.14 | 0.60, 2.17 |  |
| Puffed food-Candy-Bakery | Q1 | Ref | - | 0.352 |  | Ref | - | 0.698 |  | Ref | - | 0.364 |
|  | Q2 | 0.57 | 0.24, 1.32 |  |  | 0.37 | 0.13, 1.10 |  |  | 0.88 | 0.46, 1.70 |  |
|  | Q3 | 1.06 | 0.52, 2.16 |  |  | 1.26 | 0.56, 2.86 |  |  | 1.05 | 0.56, 1.95 |  |
|  | Q4 | 0.50 | 0.20, 1.25 |  |  | 0.99 | 0.36, 2.69 |  |  | 1.29 | 0.68, 2.47 |  |
| Dried fruits-Organs-Rice | Q1 | Ref | - | 0.359 |  | Ref | - | 0.087 |  | Ref | - | 0.750 |
|  | Q2 | 1.48 | 0.64, 3.40 |  |  | 1.60 | 0.58, 4.43 |  |  | **2.68** | **1.41, 5.11**** |  |
|  | Q3 | 1.23 | 0.52, 2.91 |  |  | **2.78** | **1.08, 7.20*** |  |  | 1.81 | 0.92, 3.56 |  |
|  | Q4 | 1.65 | 0.68, 3.98 |  |  | 2.33 | 0.77, 7.09 |  |  | 1.25 | 0.59, 2.65 |  |

^a^ Models adjusted for age, depression status, the supplement of multi-vitamins and vitamin B, and total energy.

^b^ Models adjusted for age, BMI, educational level, stress, physical activity, work intensity, the supplement of folic acid, vitamin A, and vitamin D, and total energy.

^c^ Models adjusted for age, BMI, smoking status, stress, sleep quality, the consumption of alcoholic and functional beverages, spicy food intake, vitamin E supplement, and total energy.

**Supplementary Table 5.** Associations of the dietary patterns derived by principal component analysis with normal fertilization and hypertensive disorders during pregnancy.

| Dietary pattern | | Normal fertilization | | |  |  | Hypertensive disorders during pregnancy ^b^ | | |
| --- | --- | --- | --- | --- | --- | --- | --- | --- | --- |
|  |  | Adjusted RR | 95% CI | *P* _for trend_ |  |  | Adjusted OR | 95% CI | *P* _for trend_ |
| **BMI < 25 kg/m^2^** |  |  |  | 0.206 |  |  |  |  | 0.919 |
| Fruits-Vegetables-Dairy-Eggs | Q1 | Ref | - |  |  |  | Ref | - |  |
|  | Q2 | **1.04** | **1.01, 1.08*** |  |  |  | 0.36 | 0.08, 1.53 |  |
|  | Q3 | 0.98 | 0.95, 1.02 |  |  |  | 0.34 | 0.07, 1.74 |  |
|  | Q4 | 0.99 | 0.95, 1.03 |  |  |  | 1.02 | 0.24, 4.39 |  |
| Fish/Seafood-Animal blood | Q1 | Ref | - | 0.982 |  |  | Ref | - | 0.779 |
|  | Q2 | 0.99 | 0.95, 1.03 |  |  |  | 0.79 | 0.19, 3.34 |  |
|  | Q3 | 1.02 | 0.98, 1.06 |  |  |  | 0.86 | 0.21, 3.52 |  |
|  | Q4 | 1.00 | 0.96, 1.03 |  |  |  | 0.77 | 0.17, 3.51 |  |
| Tubers-Beans-Cereals | Q1 | Ref | - | **0.020*** |  |  | Ref | - | 0.541 |
|  | Q2 | 1.01 | 0.98, 1.05 |  |  |  | 2.24 | 0.54, 9.26 |  |
|  | Q3 | 1.02 | 0.98, 1.06 |  |  |  | 1.25 | 0.24, 6.67 |  |
|  | Q4 | **0.96** | **0.92, 1.00*** |  |  |  | 2.20 | 0.40, 12.14 |  |
| Puffed food-Candy-Bakery | Q1 | Ref | - | 0.877 |  |  | Ref | - | 0.697 |
|  | Q2 | 0.99 | 0.95, 1.02 |  |  |  | 1.51 | 0.35, 6.53 |  |
|  | Q3 | 1.02 | 0.98, 1.06 |  |  |  | 1.68 | 0.39, 7.32 |  |
|  | Q4 | 1.00 | 0.96, 1.04 |  |  |  | 1.44 | 0.25, 8.20 |  |
| Dried fruits-Organs-Rice | Q1 | Ref | - | 0.239 |  |  | Ref | - | 0.622 |
|  | Q2 | **0.95** | **0.91, 0.98**** |  |  |  | 0.75 | 0.17, 3.42 |  |
|  | Q3 | 1.00 | 0.96, 1.03 |  |  |  | 1.25 | 0.32, 4.78 |  |
|  | Q4 | 0.96 | 0.93, 1.00 |  |  |  | 1.35 | 0.28, 6.59 |  |
| **BMI ≥ 25 kg/m^2^** |  |  |  |  |  |  |  |  |  |
| Fruits-Vegetables-Dairy-Eggs | Q1 | Ref | - | **< 0.001**** |  |  | Ref | - | 0.194 |
|  | Q2 | **1.11** | **1.05, 1.18**** |  |  |  | 2.72 | 0.51, 14.65 |  |
|  | Q3 | **1.12** | **1.05, 1.18**** |  |  |  | **5.78** | **1.17, 28.58*** |  |
|  | Q4 | **1.17** | **1.10, 1.24**** |  |  |  | 3.67 | 0.61, 22.02 |  |
| Fish/Seafood-Animal blood | Q1 | Ref | - | 0.088 |  |  | Ref | - | **0.047*** |
|  | Q2 | 0.98 | 0.92, 1.04 |  |  |  | 0.51 | 0.16, 1.65 |  |
|  | Q3 | 0.97 | 0.92, 1.03 |  |  |  | **0.14** | **0.03, 0.73*** |  |
|  | Q4 | 0.99 | 0.93, 1.05 |  |  |  | 0.40 | 0.12, 1.33 |  |
| Tubers-Beans-Cereals | Q1 | Ref | - | **0.034*** |  |  | Ref | - | 0.847 |
|  | Q2 | **0.90** | **0.85, 0.96**** |  |  |  | 1.64 | 0.44, 6.09 |  |
|  | Q3 | 0.95 | 0.90, 1.01 |  |  |  | 1.29 | 0.34, 4.91 |  |
|  | Q4 | **0.94** | **0.89, 1.00*** |  |  |  | 1.31 | 0.31, 5.49 |  |
| Puffed food-Candy-Bakery | Q1 | Ref | - | 0.279 |  |  | Ref | - | 0.242 |
|  | Q2 | 0.98 | 0.93, 1.05 |  |  |  | 0.68 | 0.21, 2.27 |  |
|  | Q3 | **0.94** | **0.89, 1.00*** |  |  |  | 0.66 | 0.21, 2.13 |  |
|  | Q4 | 0.97 | 0.91, 1.03 |  |  |  | 0.42 | 0.10, 1.83 |  |
| Dried fruits-Organs-Rice | Q1 | Ref | - | 0.776 |  |  | Ref | - | 0.725 |
|  | Q2 | 1.03 | 0.97, 1.09 |  |  |  | 1.16 | 0.32, 4.21 |  |
|  | Q3 | 1.02 | 0.96, 1.08 |  |  |  | 2.26 | 0.69, 7.43 |  |
|  | Q4 | 1.01 | 0.95, 1.08 |  |  |  | 0.99 | 0.20, 4.94 |  |

^a^ Models adjusted for age, BMI, the type and cause of infertility, insemination method, depression status, sleep quality, physical activity, alcoholic beverages consumption, spicy food intake, the supplement of vitamin D, calcium, and iron, and total energy.

^b^ Models adjusted for age, BMI, type and cause of infertility, physical activity, the supplement of vitamin A and vitamin D, and total energy. 20 women in a state of ongoing pregnancy were excluded from the analysis.

1. **Supplementary Figures**

**
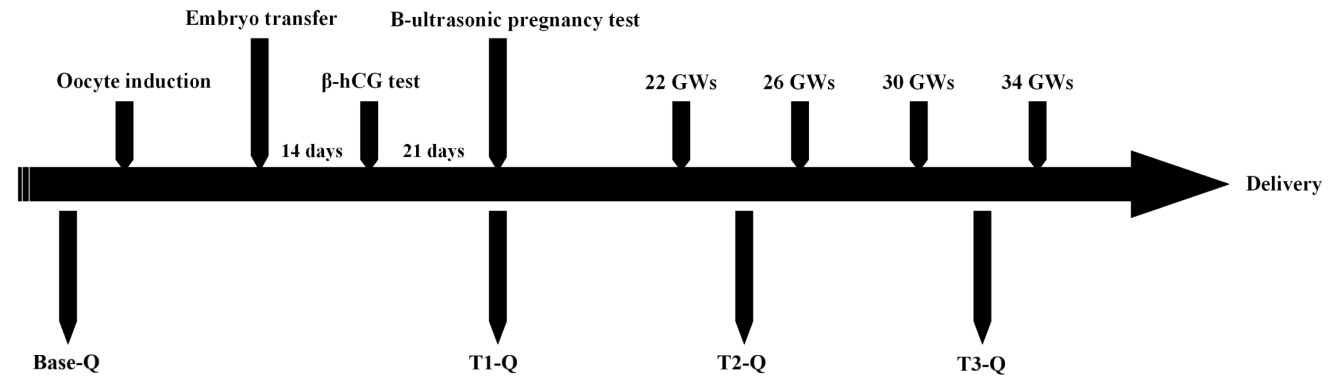
**

**Supplementary Figure 1.** Timeline of questionnaire collection.


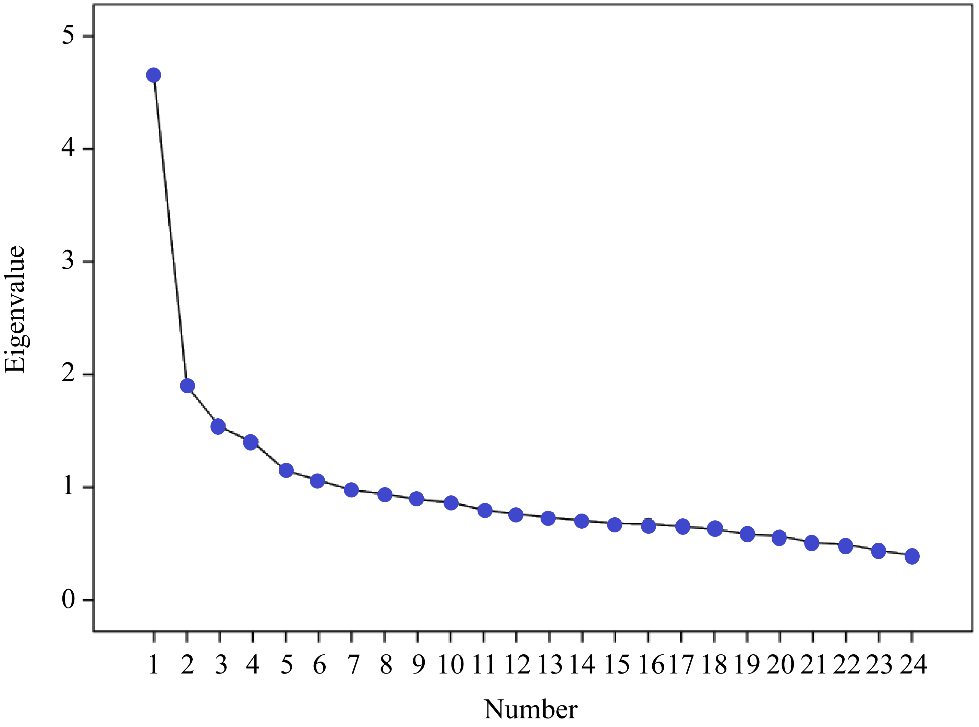


**Supplementary Figure 2.** Scree plot for identification of dietary patterns by principal component analysis. Components with an eigenvalue >1 were retained.
